# *YAP1::KMT2A*‐Rearranged Sarcoma: Report of a New Case With Unusual Morphology and Immunohistochemical Features

**DOI:** 10.1002/gcc.70059

**Published:** 2025-07-11

**Authors:** Caterina Fumagalli, Ruth Orellana, Sílvia Bagué, Malena Ferré, Allan Gonzalez, Lluis Catasús, Jaume Llauger, Ana Peiró, Paul Zamora Alarcón, Katarina Majercakova, Raúl Terés, Marie Karanian‐Philippe, Franck Tirode, Cristina R. Antonescu

**Affiliations:** ^1^ Department of Pathology Hospital de la Santa Creu i Sant Pau Barcelona Spain; ^2^ Department of Radiology Hospital de la Santa Creu i Sant Pau Barcelona Spain; ^3^ Department of Traumatology and Orthopedics Hospital de la Santa Creu i Sant Pau Barcelona Spain; ^4^ Department of Plastic and Reconstructive Surgery Hospital de la Santa Creu i Sant Pau Barcelona Spain; ^5^ Department of Radiation Oncology Hospital de la Santa Creu i Sant Pau Barcelona Spain; ^6^ Department of Oncology Hospital de la Santa Creu i Sant Pau Barcelona Spain; ^7^ Department of Pathology Centre Léon Bérard, Centre de Recherche en Cancérologie de Lyon, INSERM U1052—CNRS UMR5286, Université de Lyon, Université Claude Bernard Lyon 1 Lyon France; ^8^ Department of Pathology and Laboratory Medicine Memorial Sloan Kettering Cancer Center New York New York USA

**Keywords:** *KMT2A::YAP1* fusion, sarcoma, sclerosing epithelioid fibrosarcoma

## Abstract

Recurrent *KMT2A* and *YAP1* related fusions have recently been reported in various mesenchymal neoplasms of different histogenesis. First, *YAP1::KMT2A* fusions have been described in a subset of MUC4‐negative sclerosing epithelioid fibrosarcomas (SEF), while *VIM::KMT2A* fusions in a handful of cases associated with an undifferentiated spindle cell phenotype lacking stromal hyalinization. On the other hand, *YAP1* gene rearrangements have been reported in a wide spectrum of sarcomas, including vascular neoplasms such as epithelioid hemangioendothelioma (EHE). Despite these molecular advances, occasional challenges in classification may occur even if the pathognomonic fusion is identified. In this study, we report such a case of a soft tissue sarcoma displaying an unusual morphology and immunoprofile, which remained unclassified even after a *YAP1::KMT2A* fusion was detected. The lesion occurred in the left leg of a 65‐year‐old female and microscopically closely resembled a SEF, with epithelioid morphology organized in cords, nests, and sheets in a heavy hyalinized background. Focally, the cells showed cytoplasmic vacuoles with eosinophilic material, reminiscent of the “blisters cells” seen in EHE. Moreover, by immunohistochemistry (IHC), the tumor showed diffuse reactivity for vascular markers, including ERG, CD31, CD34, and D2‐40, as well as for TFE3, while being negative for MUC4, CAMTA1, smooth‐muscle actin, desmin, S100 and keratins. Targeted RNA sequencing revealed a *YAP1::KMT2A* fusion. Based on this molecular result and the conflicting morphologic and IHC findings, a definitive distinction between a MUC4‐negative SEF and an EHE could not been established. To further subclassify the lesion, subsequent clustering analysis using RNAseq signature was performed against a vast group of sarcoma types on the same array. Results showed that the tumor was in close proximity to the SEF group, admixed together with the other *YAP1::KMT2A* MUC4 negative SEF sarcomas. This case is highly instructive, as it shows another application of RNA sequencing in clinical practice when discordant or uncertain results between pathologic findings and fusion type may occur. Indeed, RNAseq signature could help, in this context, to better classify the tumor as a *YAP1::KMT2A* sarcoma instead of a vascular tumor. Larger series are needed to evaluate the pathogenesis of these tumors and the relevance of vascular markers expression.

## 1 Introduction

In the last decade, due to an expanding application of targeted RNA sequencing in clinical practice, new entities or molecular variants of known sarcomas have emerged based on novel genetic alterations. One such example is the *YAP1::KMT2A* fusions, which have coined a subset of sclerosing epithelioid fibrosarcomas that were negative for MUC4 expression but otherwise displayed a classic morphology [1, 2, 3, 4, 5]. Shortly after, a variant *VIM::KMT2A* fusion was reported in a handful of cases with a somewhat distinct microscopic appearance characterized by an undifferentiated round to spindle phenotype lacking hyalinization and often following an aggressive clinical course [2, 6]. Moreover, *YAP1*‐associated fusions have been described in a large range of neoplasms, including supratentorial ependymomas [7], poromas/porocarcinomas [8] and, within soft tissue tumors, a subset of epithelioid hemangioendothelioma (EHE) [9, 10] and a recently described variant of myxoinflammatory fibroblastic sarcoma [11]. The present study describes an unusual soft tissue sarcoma with a morphology consistent with SEF but an immunoprofile suggestive of endothelial differentiation. Despite the identification of a *YAP1::KMT2A* gene fusion by NGS, the differential diagnosis remained between SEF and EHE. Subsequent clustering analysis using RNAseq signature was performed, showing that the tumor was in close proximity to the SEF group, admixed together with the other *YAP1::KMT2A*‐ rearranged sarcomas, excluding its clustering with EHE. We present this case in order to report the atypical immunoprofile of the tumor and to highlight the contribution of additional genomic assays in assessing a final classification.

## 2 Material and Methods

In the clinical work‐up of a sarcoma with epithelioid morphology, targeted RNA sequencing was performed for a more definitive subclassification due to unusual and conflicting histologic findings and immunoprofile. The molecular results revealed a *YAP1::KMT2A* gene fusion, prompting an in‐depth review of the morphologic features and immunophenotype, as well as further genomic studies, such as RNAseq clustering analysis. Follow‐up information was available. Informed consent was obtained from the patient reported in this study.

### 2.1 Immunohistochemistry

Immunohistochemical stains using the following commercially available antibodies were performed using [Dako OMNIS]: CAMTA1 (Clone Polyclonal Gennova, 1:100), CD31 (Clone JC70A, Dako, RTU), CD34 (Clone QBEnd 10, Dako, RTU), CK AE1/AE3 (Clone AE1/AE3, Dako, RTU), D2‐40 (Clon D2‐40, Dako, RTU), Desmin (Clone D33, Dako, RTU), ERG (Clone EP111, Dako, RTU), MUC‐4 (Clone SP241, Gennova, 1:25), SATB2 (Clone EP281, Gennova, 1:100), SMA (Clone 1A4, Dako, RTU), S100 (Clone Polyclonal, Dako, RTU), SMARCB1 (Clone 25/BAF47, Gennova, 1:50) and TFE3 (Clone EPR11591, Gennova, 1:100). All positive and negative controls showed appropriate staining.

### 2.2 RNA Sequencing

RNA was extracted from formalin‐fixed paraffin‐embedded (FFPE) tissue using the AllPrep DNA/RNA FFPE Kit (Qiagen GmbH, Hilden, Germany) as specified by the manufacturer's instructions. The case was tested by Anchored Multiplex PCR (AMP) sequencing assay using the Archer FusionPlex Expanded Sarcoma panel (ArcherDX, Boulder, CO) according to the manufacturer's instructions. Briefly, the library was created by using the FusionPlex assay in conjunction with the Gene specific primers (GSPs) and Archer Molecular Barcode Adapters (MBCs) for Illumina. The library was sequenced on the Illumina MiSeq (Illumina, San Diego, Ca), and the data was analyzed using the Archer software (Suite_Analysis_v6.2.3) to detect and identify partners of 55 genes reported to be associated with soft tissue tumors.

### 2.3 Whole‐Exome RNA Sequencing and Clustering Analysis

Whole‐exome RNA sequencing (WERS), expression profile analyses including several clustering methods, detection of fusion genes, and small nucleotide variations have been performed at the Centre Leon Bérard (CLB) in Lyon using exome‐based RNA capture sequencing on the archival material, as previously described [12].

## 3 Results

### 3.1 Case Report

**FIGURE 1:**
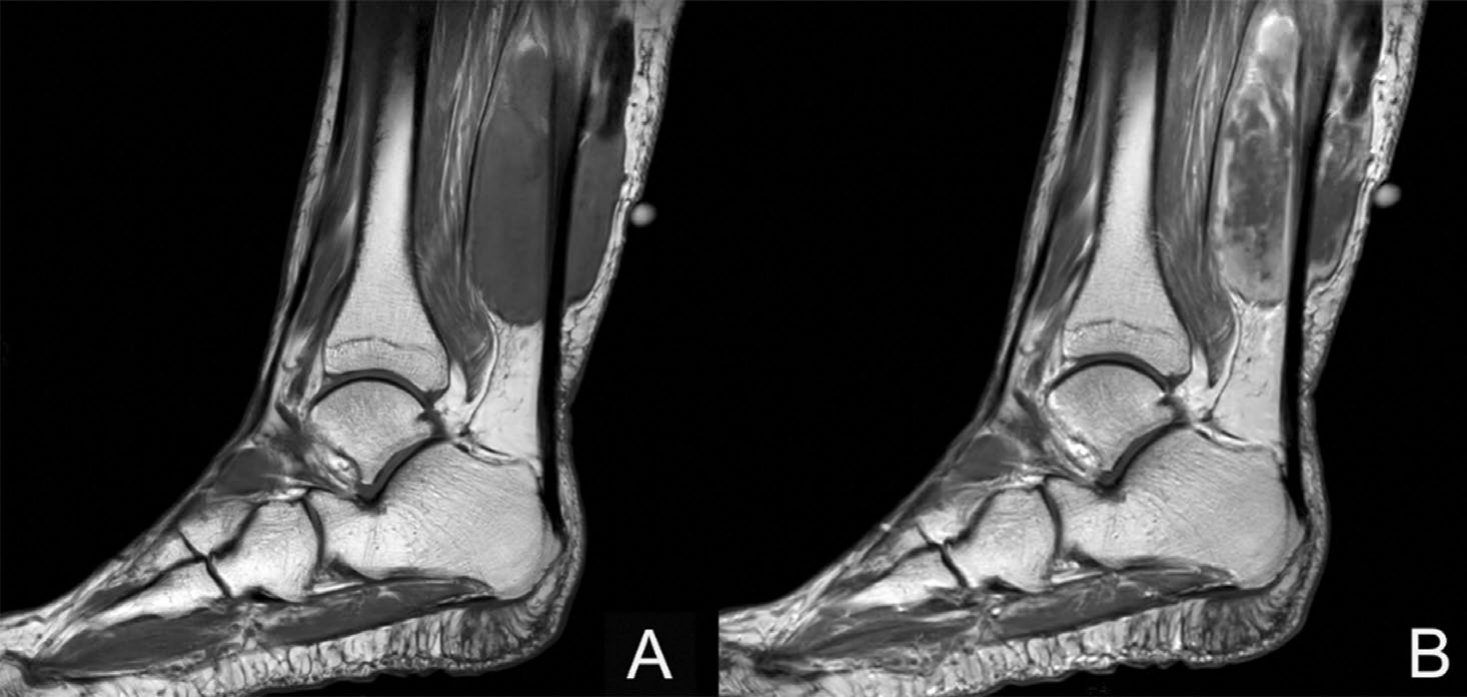
MRI showing a deep intramuscular mass related to the Achilles tendon. The tumor was hypointense in T1 sequence (A), with heterogeneous enhancing after contrast administration (B).

The patient, a 64‐year‐old female with no significant previous medical history, presented to our institution with a painful left leg mass. Diagnostic MRI revealed a well‐defined tumor measuring 9 × 5 × 4 cm and located in the gastrocnemius muscle with Achilles tendon's involvement. The tumor was isointense in T1‐weighted imaging and hyperintense and heterogeneous in T2‐weighted imaging, with a cranial hypointense region suggestive of fibrosis, calcification, or chronic hemorrhage. After contrast administration, an intense and heterogeneous enhancement was present (Figure 1).

**FIGURE 2:**
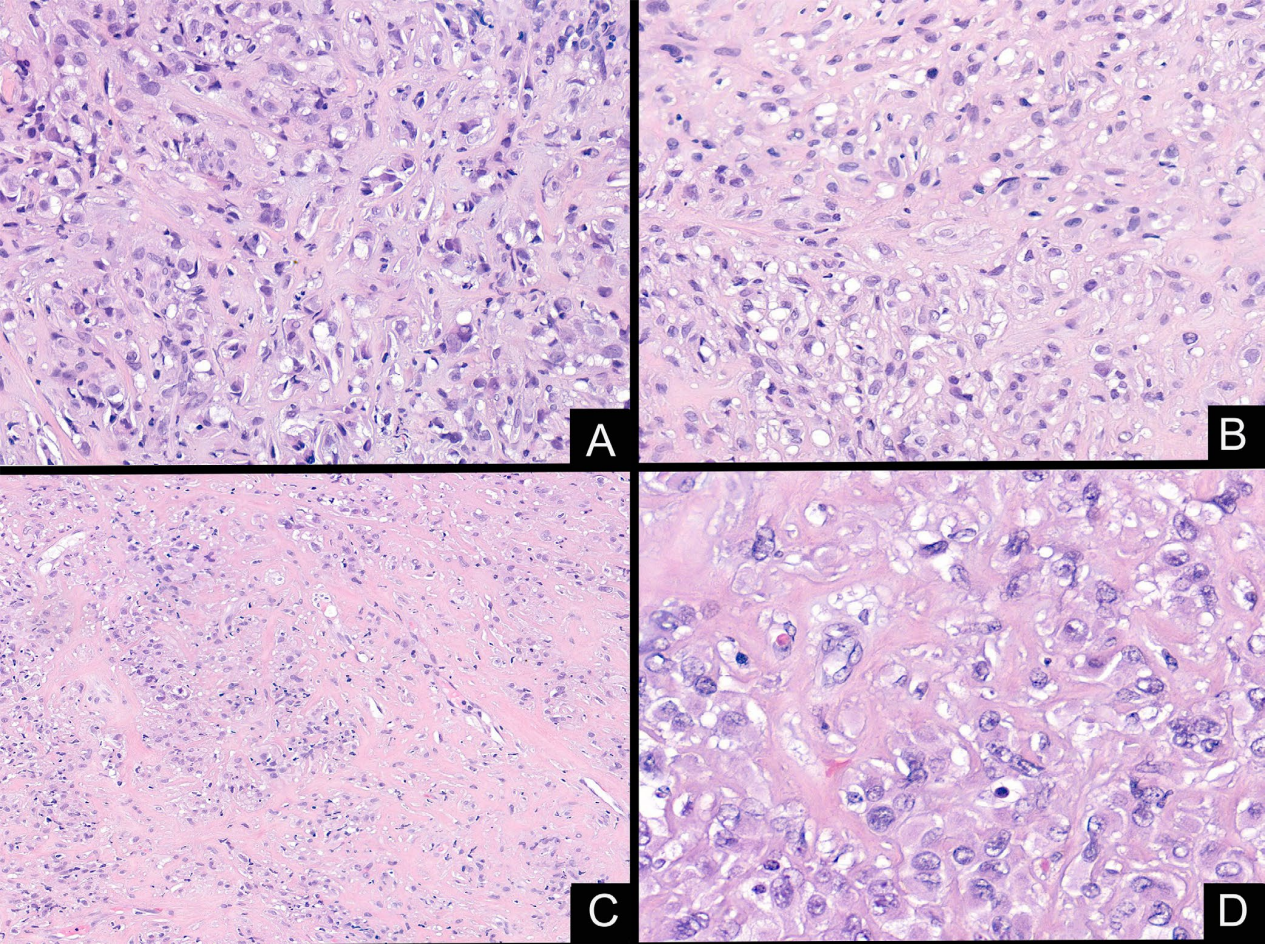
Low power view of the initial biopsy showing neoplastic cells with epithelioid morphology and focal cytoplasmic vacuolization (A), embedded in a myxo‐hyaline stroma (B). Areas with a dense collagen background were seen (C). At high power, isolated cells showed vacuolated cytoplasm containing eosinophilic material resembling erythrocytes (D).

The mass was biopsied, showing a cellular proliferation of predominantly epithelioid cells, with eosinophilic cytoplasm and ovoid nuclei with fine chromatin. Tumor cells were arranged in cords, nests and in sheets, within a hyalinized and focally myxoid stroma. Isolated cells presented a cytoplasmic vacuolization with an eosinophilic material within (Figure 2). The tumor had a low mitotic rate (up to one mitosis in 10 high power fields) and small foci of necrosis were found. Immunohistochemically, the neoplastic cells were diffusely positive for CD31, CD34 and ERG. MUC4, CAMTA1, Keratin AE1/AE3 and S100 were negative. INI‐1 expression was preserved.

A provisional diagnosis of “sarcoma with epithelioid morphology and vascular markers expression” was made and the patient was referred to the multidisciplinary sarcoma board, with the decision of surgery followed by radiotherapy depending on final diagnosis and margins status. A pre‐surgery evaluation with abdominal and thoracic CT showed a non‐specific 4 mm pulmonary nodule localized in the left lower lobe.

**FIGURE 3:**
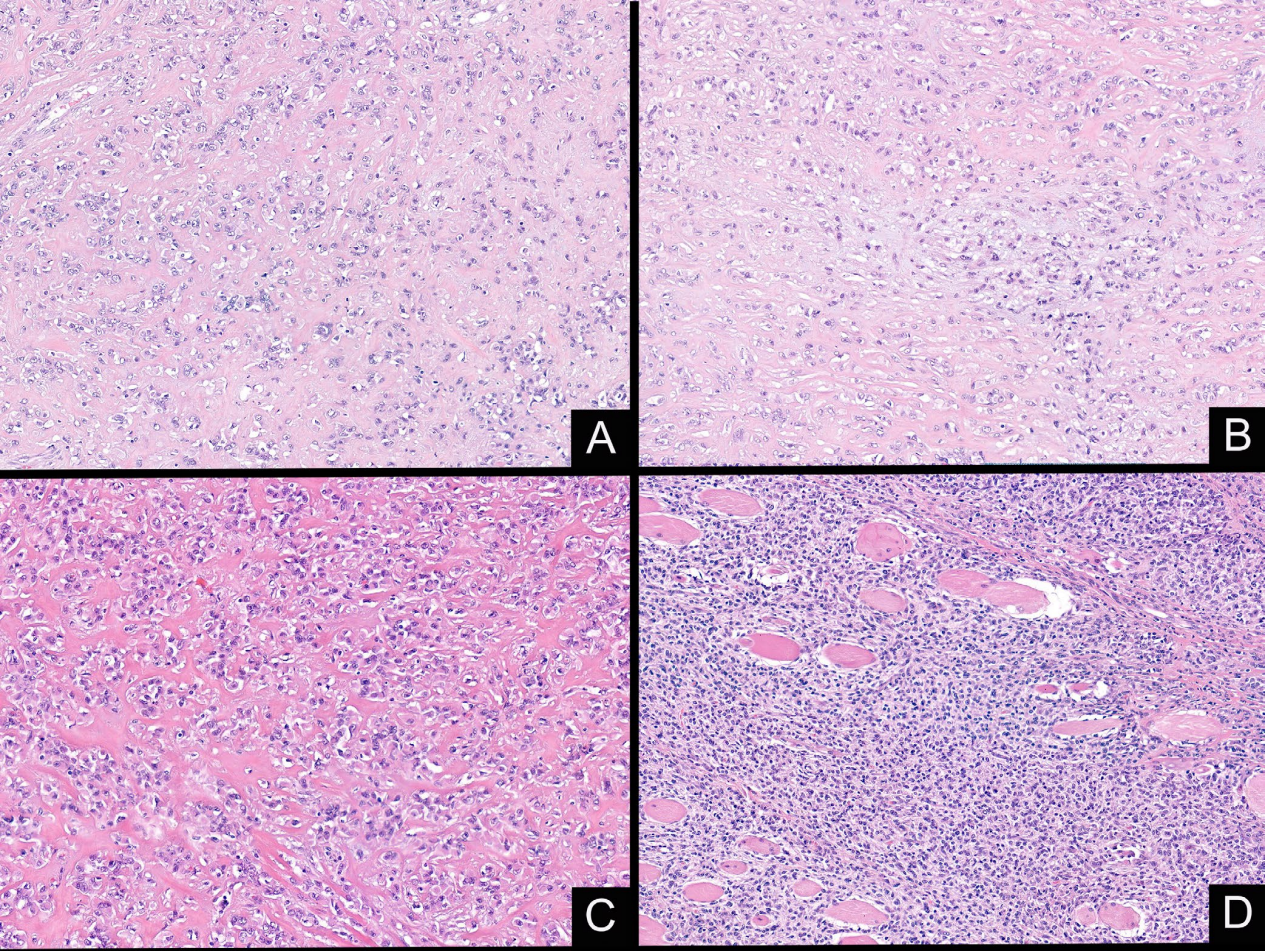
Resection specimen presented areas with monomorphic epithelioid neoplastic cells showing focal cytoplasmic vacuolization embedded in a myxohyaline stroma, similar to that seen in the initial biopsy (A and B.); tumor cells arranged in nests and cords are embedded in a dense collagen background (C); areas with a more primitive round cell proliferation and infiltrative margins were identified (D).

The surgical specimen consisted of a well‐defined mass involving the Achilles tendon, with a firm white‐gray cut surface. Histologically, the tumor showed a variegated phenotype. Most of the lesion showed a hypocellular proliferation of monomorphic epithelioid cells arranged in cords and single files, embedded in a densely hyalinized collagenous stroma. These areas were closely reminiscent to an SEF‐like pattern. In addition, as observed in the initial biopsy, certain areas of the tumor resembled more an EHE, with vacuolated epithelioid cells containing eosinophilic inclusions within a fibromyxoid background. Furthermore, the tumor included areas with a more primitive small blue round cells and rhabdoid morphology, with scant eosinophilic cytoplasm, and eccentric nuclei (Figure 3). The tumor had a low mitotic rate, up to four mitoses in 10 high power fields. Foci of necrosis (less than 50% of tumoral volume) were identified in the center of the mass.

**FIGURE 4:**
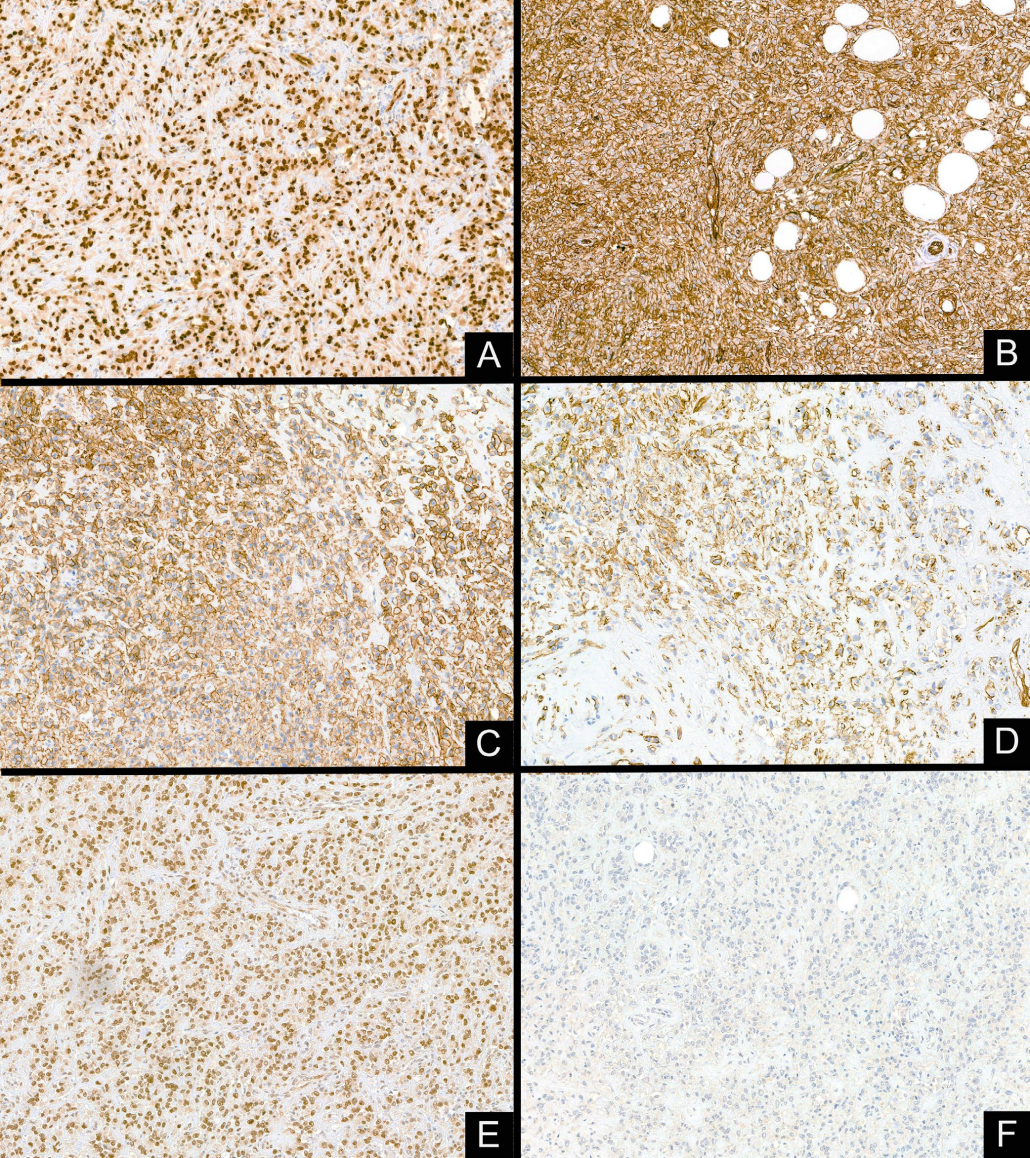
The immunohistochemical study showed that the tumoral cells were strong and diffusely positive for ERG (A), CD31 (B), and podoplanin (C), with patchy expression of CD34 (D). Furthermore, TFE3 positivity was present (E). MUC4 was negative (F).

The immunophenotype was consistent with the initial biopsy, showing diffuse and strong positivity for ERG, CD31, and patchy expression of CD34, along with a lack of MUC4 expression. Additional studies showed positivity for podoplanin and TFE3 (Figure 4). Vascular marker reactivity was noted in both EHE‐like areas and SEF‐like zones.

**FIGURE 5:**
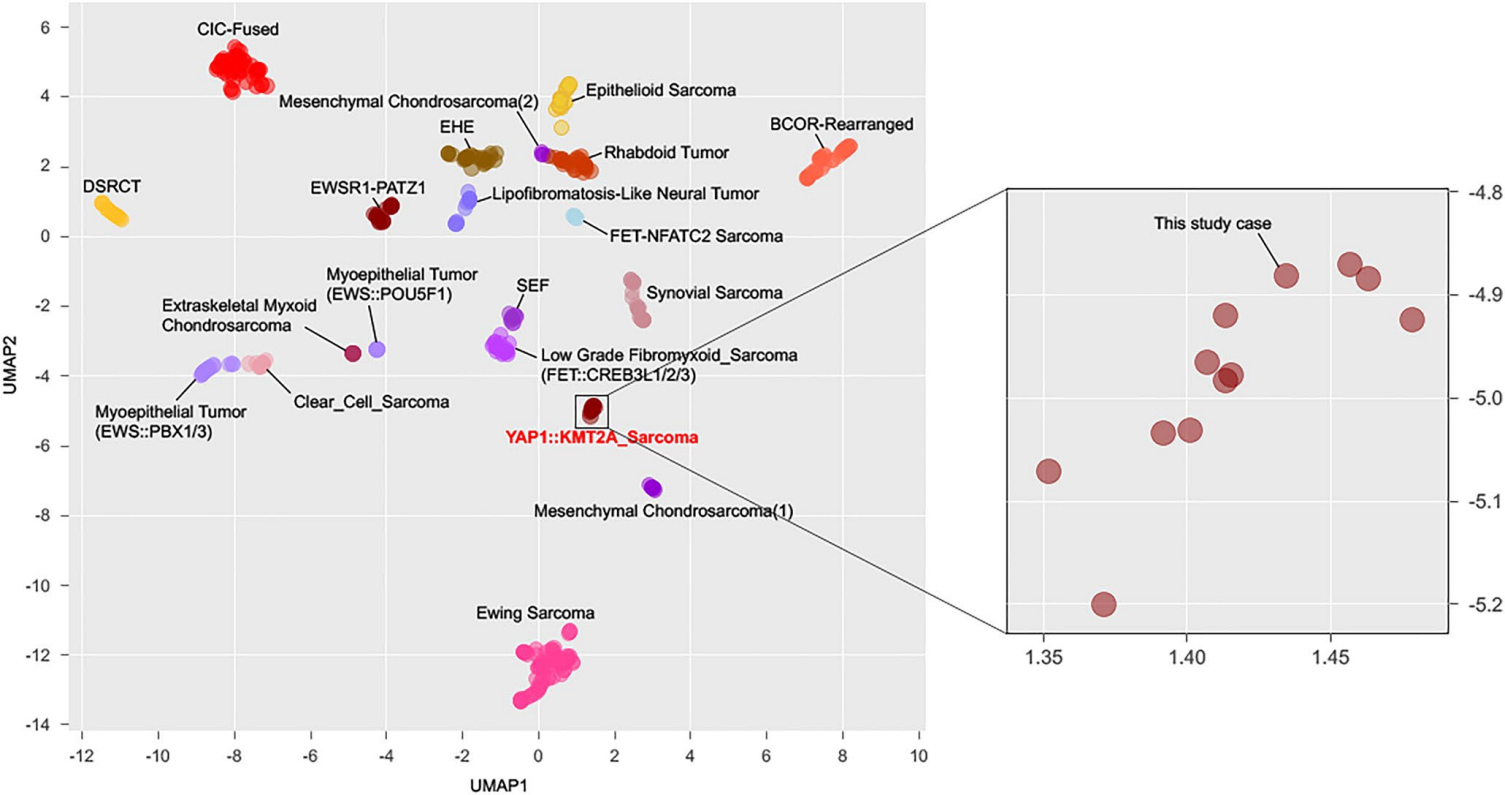
Clustering analysis highlighted that the reported case clustered with the other YAP1::KTM2A sarcomas, when compared to a variety of other soft tissue or bone tumors in a Uniform Manifold Approximation and Projection (UMAP) analysis.

Subsequent Anchored Multiplex RNA sequencing assay using the Archer FusionPlex Expanded Sarcoma panel revealed a *YAP1::KMT2A* fusion and a reciprocal *KMT2A::YAP1* fusion transcript. The first rearrangement corresponded to *YAP1* exon 4 (chr11:102076817) fused with *KMT2A* exon 5 (chr11:118347520), while the second product was generated for the fusion between *KMT2A* exon 6 (chr11:118350953) and *YAP1* exon 9 (chr11:102100433). The tumor was finally diagnosed as a *YAP1::KMT2A* sarcoma. Due to the conflicting immunohistochemical profile and the morphology EHE‐like, RNAseq and subsequent clustering analysis were performed, revealing that the tumor clustered with the other *YAP1::KMT2A* sarcomas, away from the EHE group and closer to SEF (Figure 5). This result aid to finally classify the tumor as a *YAP1::KMT2A* MUC4 negative SEF sarcoma.

The patient started adjuvant radiotherapy (68 Gray) following marginal excision. A computed tomography scan performed 15 months after surgery showed an enlarging pulmonary nodule and two additional intrapulmonary lesions. All were surgically resected and confirmed to be metastatic disease. Histology of the metastatic sites revealed features consistent with the primitive small round cell morphology described in the surgical specimen. The mitotic rate was higher than the primary tumor (up to seven mitoses in 10 high power field) and no necrosis was found. The patient is now free of local recurrence after 24 months from primary surgery, but continues to have metastatic pulmonary disease.

## 4 Discussion

Gene fusions involving *lysine methyltransferase 2A* (*KMT2A*) gene, previously known as *mixed‐lineage leukemia* (*MLL*), have been extensively implicated in the pathogenesis of acute myeloid leukemias in association with a wide range of gene partners [13, 14]. More recently, Watson et al. first described a *KMT2A::YAP1* fusion in a spindle cell sarcoma resembling fibrosarcoma with a sclerosing background in a 35‐year‐old female, which was detected during a large RNA‐sequencing screening of 185 small round cell sarcomas [4]. Subsequently, Yoshida et al. described two additional cases of *KMT2A*‐rearranged soft tissue sarcomas (one each with *YAP1* and *VIM* gene partners) occurring in young adults and following an aggressive clinical course [5]. Additional cases of *KMT2A*‐rearranged sarcomas, including two series of eight and nine patients, respectively, describing recurrent fusions between *YAP1* and *KMT2A* genes in a group of sarcomas resembling morphologically a sclerosing epithelioid fibrosarcoma with negativity for MUC4 and absence of alterations in *FUS* or *EWSR1* genes have been published, leading to a better characterization of these sarcomas [1, 2, 3, 6, 15, 16].

*YAP1::KMT2A* sarcomas typically affect middle‐aged patients (median age of 45 years) of both genders and involved somatic soft tissues with a wide distribution. Morphologically, the tumors show infiltrative margins, variable cellularity, and an SEF‐like morphology, consisting of monomorphic round‐to‐epithelioid cells arranged in sheets or cords within a sclerotic stroma. Mitoses and necrosis may be variably present within the tumors.

On the other hand, *VIM::KMT2A* sarcoma shows a similar median age at diagnosis but a male predominance, along with a wide distribution both in soft tissue and visceral localization [2, 6]. Histologically, the tumors show a more uniform hypercellularity of round, ovoid to short spindle cells arranged in fascicular and storiform patterns, in the absence of collagenous and hyalinized stroma typically seen in the *YAP1::KMT2A* sarcomas.

Furthermore, a recent case of *KMT2A‐*rearranged sarcoma occurring in the right supraclavicular region of a 47‐year‐old man with an unbalanced, three‐way fusion, *CBX6::KMT2A::PYGO1*, has been reported [17]. Morphologically, the tumor consisted of small round‐to‐spindle cells arranged in short fascicles and embedded in variable collagenous and sclerotic stroma, with a high mitotic index.

A variable and non‐specific immunohistochemical profile has been so far described across *KMT2A*‐rearranged sarcomas, as the patchy expression of CD99, EMA, ERG, CD31, CD34, BCOR, and S100 was found in some but not consistently in most tumors. However, all cases tested were negative for MUC4. Regarding vascular markers, their expression was found somewhat contradictory: in the series published by Puls et al. [3] there was no expression found, while in the series of Kao et al. [1] focal ERG and CD34 were described in one out of three and three out of seven tested cases, respectively. As in most instances, a vascular tumor was not considered in the differential diagnosis; therefore, vascular markers were not assessed in all cases.

In this report, we describe a soft tissue sarcoma with epithelioid morphology, harboring a *YAP1::KMT2A* and a *KMT2A::YAP1* fusion products detected by Archer FusionPlex Expanded panel. The morphologic appearance showed hybrid features: some areas displayed an abundant collagenous background reminiscent of SEF, while others contained vacuolated epithelioid cells embedded in a fibromyxoid stroma, resembling EHE. Moreover, by immunohistochemistry, there was diffuse and strong positivity for all vascular markers tested, including ERG, CD31, and CD34, as well as for TFE3 and podoplanin, while MUC4 was negative. Although some patchy endothelial marker expression has been reported in *YAP1::KMT2A* sarcomas, none of the cases showed diffuse and strong expression as in our case.

Due to the conflicting pathology‐molecular correlation and the suspicion of a vascular origin, an unsupervised RNA sequencing clustering of a vast cohort of soft tissue sarcomas was applied as an additional tool to resolve this classification dilemma. Results showed unequivocal clustering of the index case closer to the entire SEF group, far from the EHE group, and within the *YAP1::KMT2A*‐positive sarcoma subset. Recently, similar results had been published by Warmke et al. [16], showing that *YAP1::KMT2A*‐rearranged sarcoma formed a distinct cluster, which was separate from SEF. The use of RNAseq gene signature as a tool for further classification has been previously applied in a few other studies due to an increasing awareness of gene fusion promiscuity in sarcomas. One such example is the large spectrum of tumors driven by *EWSR1::CREB* fusions, where occasional morphologic and immunohistochemical overlap may result in classification challenges. Thus, in a prior study, we have shown that gene expression clustering is based on tumor type and not fusion type [18]. Moreover, within this family of tumors, the recently described group of malignant epithelioid neoplasms with a predilection for serosal surfaces, showing hybrid features of angiomatoid fibrous histiocytoma and mesothelioma, by RNAseq clustered separate from other entities sharing *EWSR1::CREB* type fusions [19]. Another example involves tumors at the crossroads between round cell sarcomas and certain molecular subtypes of myoepithelial tumors, where additional RNA sequencing clustering may provide further support in classification [20].

Additional studies are needed to determine if this aberrant vascular marker expression represents an isolated finding within this family of tumors or is an indication for a more consistent divergent differentiation to an endothelial lineage. In keeping with this observation, is the expression of CD31 and ERG markers in a small subset of *CIC‐*rearranged sarcomas, which have initially regarded as poorly differentiated/round cell angiosarcoma [21], however, subsequently shown, also by RNA sequencing clustering, that they group together with most other *CIC*‐positive sarcomas [22].

In summary, we are reporting a remarkable case of a *YAP1::KMT2A*‐positive sarcoma showing hybrid histology and endothelial differentiation, which was best classified subsequent to RNA sequencing clustering together with the other *YAP1::KMT2A*‐rearranged sarcomas, highlighting the role of additional/complementary molecular testing to achieve a final subclassification.

## Ethics Statement

This study was performed following institutional REB approval.

## Consent

Informed consent was obtained from the patient reported in this study.

## Conflicts of Interest

The authors declare no conflicts of interest.

## Data Availability

The data that support the findings of this study are available on request from the corresponding author. The data are not publicly available due to privacy or ethical restrictions.
